# MIAuSport: a feasibility and acceptability study of a workplace-based physical activity program for hospital healthcare workers

**DOI:** 10.3389/fspor.2026.1782609

**Published:** 2026-09-18

**Authors:** Jean Regina, Claire Seydoux, Clémentine Grand, Vincent Gremeaux, Peter Vollenweider, Vanessa Kraege, Roxane De La Harpe, Denis Comte

**Affiliations:** 1 Internal Medicine Division, Department of Internal Medicine, University Hospital of Lausanne and Lausanne University, Lausanne, Switzerland; 2 Communication Division, Department of Internal Medicine, University Hospital of Lausanne, Lausanne, Switzerland; 3 Department of Sports Medicine, Swiss Olympic Medical Center, Lausanne University Hospital, Lausanne, Switzerland; 4 Institute of Sports Sciences, University of Lausanne, Lausanne, Switzerland; 5 Innovation and Clinical Research Division, Department of Internal Medicine, University Hospital of Lausanne, Lausanne, Switzerland

**Keywords:** acceptability, feasibility, healthcare worker, implementation, physical activity, Switzerland, workplace

## Abstract

**Objectives:**

Physical activity (PA) is a fundamental determinant of health. Healthcare workers (HCWs) play a key role in PA promotion but often face high workloads and organizational barriers limiting their own PA. Workplace programs may provide an opportunity to improve PA levels in this population. This study describes the feasibility and acceptability of *MIAuSport*, a workplace-based PA program for internal medicine residents in a Swiss University Hospital.

**Material and methods:**

*MIAuSport* consists of a 40-minute outdoor session held monthly during lunch time hours, offering walking and running options. The program was implemented from February 2024 to January 2025. Attendances were recorded anonymously at each of the 12 sessions. Anonymous post-session and eight-month surveys were used to measure feasibility, acceptability, awareness, penetration, and implementation barriers and facilitators.

**Results:**

Across 12 sessions, 231 attendances were recorded, 79% of which were medical staff. The average number of attendances was 19 per session. Post-session surveys (54% response rate) indicated high acceptability, with 97% of respondents rating the organization as satisfactory, 96% finding the activity beneficial, and 84% reporting feeling motivated to increase their PA. The eight-month survey (66% response rate) showed that 88.6% (31/35) of respondents were aware of the program but only 59.4% (19/32) participated at least once in the PA program, indicating high awareness but moderate penetration. Main barriers included workload, scheduling, and weather.

**Conclusions:**

*MIAuSport* proved feasible and well received among HCWs, though sustained individual engagement could not be assessed and remains challenging.

## Introduction

Physical activity (PA) is a fundamental determinant of health, and its promotion is a key priority of the World Health Organization's (WHO) action plan for a healthier world by 2030 (1). Back in 2010, the WHO already estimated that 6% of global mortality could be attributed to physical inactivity (2). Multiple studies have shown that healthcare workers (HCWs) play an important role in promoting PA among their patients, making PA counseling a growing public health priority (3, 4).

Research further shows that HCWs who are physically active themselves are significantly more likely to encourage PA to their patients, and that patients are more likely to follow their advice (5–9). Despite recognizing the importance of PA, HCWs continue to experience alarmingly high rates of poor mental and physical health, often placing a low priority on maintaining healthy lifestyle behaviors (10). In the United Kingdom, 42% of doctors do not meet international PA recommendations (11), and a meta-analysis reported that 25% of nurses never engaged in personal PA (12). This sedentary lifestyle is influenced by multiple factors, including heavy workloads, irregular schedules, limited free time, and a broader healthcare system that offers few initiatives to promote PA and support well-being among HCWs (10, 13, 14).

Given that individuals spend on average almost half of their waking hours at work, the workplace represents an ideal setting for promoting daily PA. A nationally representative study of HCWs in the United States found that the availability of workplace PA programs was significantly associated with meeting WHO guidelines (15). Additionally, those who engage in PA during work hours are more likely to remain active outside of work (15). Workplace PA has also been linked to improved well-being, enhanced social interactions, reduced burn-out, and lower perceived physical exertion (16–18). Importantly, HCWs themselves show a strong willingness to be active, with 90% indicating that they would participate in daily exercise if given the opportunity (14).

Even in demanding professional environments, PA can be promoted through practical and adaptable interventions. While the WHO suggests at least 150 min of moderate-intensity PA or 75 min of vigorous-intensity PA per week for adults (19), as well as regular strength training, they also emphasize taking advantage of every opportunity to move throughout the day, with the slogan “every move counts towards better health” (20). The goal is to integrate PA into daily routines, regardless of individual physical condition or time constraints. This emerging paradigm is supported by studies showing that even minimal amounts of daily PA - regardless of duration - can significantly reduce the risk of premature all-cause and cardiovascular mortality (21–24).

To address this need, we developed the *MIAuSport* program, a PA initiative for junior and senior internal medicine residents at a Swiss University Hospital. It consists of a 40-minute monthly outdoor session held during working hours. This feasibility and acceptability study describes the program's structure, its evolution over 12 sessions within one year and the communication strategies employed. We report attendance data, respondent demographics, attendance rates, activity data, perceived motivation, and satisfaction. Additionally, the study evaluates overall program awareness and participation among all physicians working in the division throughout the year of implementation.

## Material and methods

### Target population

The PA program was implemented in the Internal Medicine Division of the Lausanne University Hospital (CHUV, Switzerland). The division includes approximately 40 junior residents (physicians in training, supervised by senior residents and attending physicians) and 12 senior residents (supervising junior residents while being overseen by attending physicians), collectively responsible for around 180 inpatients (with median occupancy rate of 90%) on the regular wards at any given time. On a typical weekday, official working hours are from 8:00 to 12:00 and 13:00 to 18:12, and 23 to 26 junior residents and 12 to 15 senior residents are present. The division is overseen by 9 attending physicians, with 6 to 7 usually on-site in the regular ward.

Additionally, the internal medicine division includes approximately 10 medical students, over 300 nurses and care staff, 10 physiotherapists, and 3 occupational therapists.

While the program was open to all HCWs of the Internal Medicine Division, it was specifically tailored to the medical staff's schedule by replacing one of the mandatory daily teaching sessions during lunchtime each month. Consequently, the primary target population for awareness and participation consisted of junior and senior medical residents. Additionally, HCWs from other CHUV departments were welcome to join the sessions voluntarily.

### Program implementation and promotion

A pilot phase (July–December 2023) assessed the program's feasibility, restructured the *MIAuSport* committee, identified logistical barriers, and helped refine the structure of the PA sessions and the communication strategy. The full *MIAuSport* program was launched in February 2024. Promotion of the program included in-person outreach by the seven members of the *MIAuSport* committee, email reminders sent to the division's medical staff two days before each session, a weeklong announcement on the Internal Medicine Division's intranet page, and inclusion in the weekly postgraduate training schedule of the Internal Medicine Division.

### Program assessment

During each session, two *MIAuSport* committee members, one leading the running group and the other the walking group, recorded the following data: session date, number of attendances in each group, number of junior and senior residents, attending physicians, medical administrative staff and other participants, duration of the workout, and average moving speed (GPS-based, excluding pauses). Attendance was recorded anonymously as aggregate counts per category, without individual identifiers; the number of unique participants could therefore not be determined. For physicians, the attendance rate was calculated by dividing the number of attendances by the total number of scheduled junior/senior residents and attending physicians the same day. Additionally, we documented weather conditions and temperature in degrees Celsius, based on measurements from MeteoSwiss, the national weather service (Lausanne station, with documentation of temperature, sunshine, and precipitation at session start time).

Following each workout session, all willing participants were invited to complete a brief anonymous online survey, accessible via a distributed QR code and via the Internal Medicine Division's intranet page. The survey collected demographic information (age and gender), participants’ workout choice (running or walking), and their evaluation of the program in terms of organization, perceived benefits, motivation, and overall satisfaction. It also assessed whether the session influenced participants to increase their general level of PA. Additionally, the survey addressed how participants became aware of the *MIAuSport* program, whether they had regular PA habits, and provided open-ended prompts for feedback on promotional strategies and for improvement suggestions (“What are your suggestions to improve the *MIAuSport* program?”). None of the questions were mandatory.

After eight months, an anonymous survey was emailed to all the original PA target population, which included 53 residents (36 junior and 17 senior) working across all regular shifts at that time. The difference in the numbers of residents participating in the session versus in the survey is explained by the different rotations within the Internal Medicine Department. The population who answered the survey at 8 months therefore does not entirely reflect the participants of the sessions. Figure 1 describes the MIAuSport participants, the once a month and the eight-month survey responders. A follow-up reminder was sent two weeks later. The survey assessed awareness (“Have you ever heard of *MIAuSport*?”), the most effective communication channel (“How did you mostly hear about it?”), participation (“How many times have you participated?” and “If never, why not?”), and the influence of regular PA habits. It also evaluated the program's impact on participants’ motivation to increase daily PA. It finally included open-ended questions on facilitators and barriers to implementation (“What could make your participation easier?”) and for non-participants (“Why have you never participated?”).

**Figure 1:**
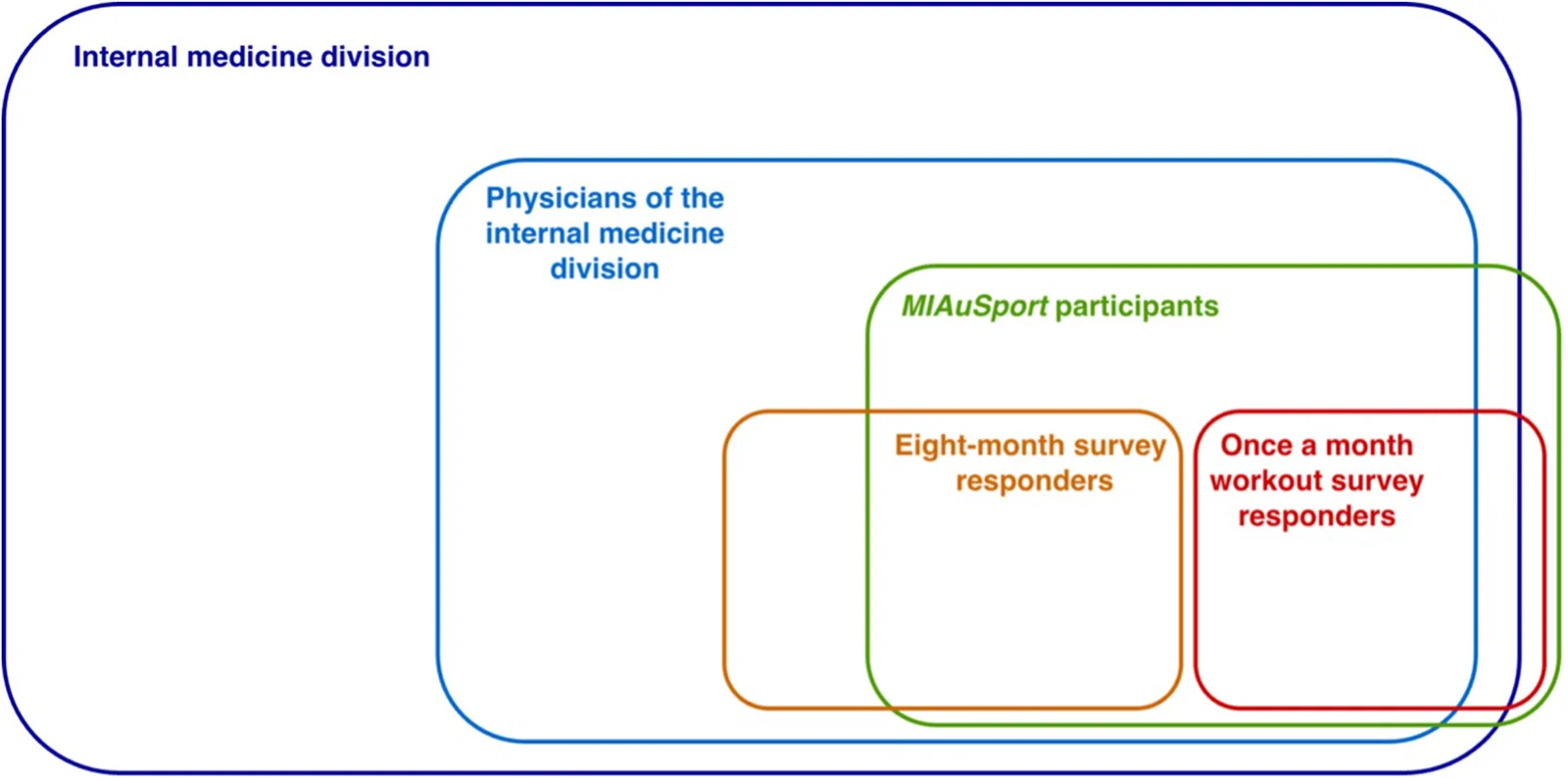
Schematic representation of *MIAuSport* and eight-month and once a month surveys responders.

Both surveys were designed and piloted by four of the authors (JR, CS, RDLH, and DC) and developed using REDCap (Research Electronic Data Capture) software, allowing for automatic export of participants’ responses to a secure database (25). Response rates and instances of incomplete surveys (i.e., at least one question answered) were recorded for each result. We did not impute missing data.

Following the implementation-outcomes taxonomy of Proctor et al. (26), we defined feasibility as the number of sessions delivered as planned and the number of session attendances per session; penetration as the proportion of eight-month survey respondents reporting at least one participation; acceptability as post-session ratings of organization, perceived benefit, and overall satisfaction; and awareness as the proportion of eight-month survey respondents aware of the program. Analyses were primarily descriptive; categorical variables are reported as counts and percentages with their denominators. Given the small subgroup sizes, no formal hypothesis testing was performed. We performed statistical analyses using the computing environment R version 4.5.0 (R Core Team, 2023) (27).

## Results

### Description of the physical activity program and its evolution over time

The PA sessions consisted of a 40-minute midday workout held once a month on Fridays. Participants could spontaneously choose between two intensity groups: walking or running. All sessions began at the hospital's main entrance at 13:00, with pre-planned routes ensuring a return by 13:40. When possible, stairs climbing was performed during the session to include a strength training and higher intensity part. Sportswear was not required, and shower facilities were available. Supplementary Table S1 outlines how the program evolved over time to better accommodate participants’ needs, particularly regarding scheduling and activity options.

### Feasibility and characteristics of the PA program and participants

Between February 2024 and January 2025, a total of 12 monthly PA sessions were conducted. During one year, a total of 231 attendances at the workout sessions were recorded (the same individual may account for several attendances), with on average 54% (125) in the walking group and 46% (106) in the running group. The mean session duration was 38.8 min for the walking group and 36.0 min for the running group. Mean speeds were 12.9 min/km for walking and 7.6 min/km for running, and mean distances covered were 3.0 km for the walking group and 4.6 km for the running group (Supplementary Table S2 for details). Characteristics of the session attendances are described in Table 1.

**Table 1:** Characteristics of session attendances stratified by activity group.

Characteristics	Overall	Walking group	Running group
Number of attendances (%)	231	125 (54.1)	106 (45.9)
Average age^a^ (SD)	36.0 ± 9.6	36.8 ± 10.6	35 ± 8.1
Gender *N* (%)			
Women (%)	60 (26.0)	46 (36.8)	14 (13.2)
Men (%)	59 (25.5)	21 (16.8)	38 (35.8)
Missing value (%)	112 (48.5)	58 (46.4)	54 (50.9)
Profession type *N* (%)			
Physician	183 (79.2)	91 (72.8)	92 (86.8)
Administrative staff	12 (5.2)	11 (8.8)	1 (0.9)
Other^b^	36 (15.6)	23 (18.4)	13 (12.3)
Physician function *N* (%)			
Junior resident	87 (47.5)	41 (45.1)	46 (50.0)
Senior resident	58 (31.7)	30 (33.0)	28 (30.4)
Attending physician	38 (20.8)	20 (22.0)	18 (19.6)

Counts refer to session attendances: the same individual may be counted more than once, and totals do not represent unique participants.

a Missing value for age for 131 attendances (56.7%).

b Others include medical students, physiotherapists from the internal medicine division, and staff from other divisions or departments.

### Attendance rate of the PA program and communication channels

Figure 2 presents weather conditions at each session and attendance rates for residents and attending physicians in the Internal Medicine Division. Across the 12 sessions, the average number of attendances per session was 19.3.

**Figure 2:**
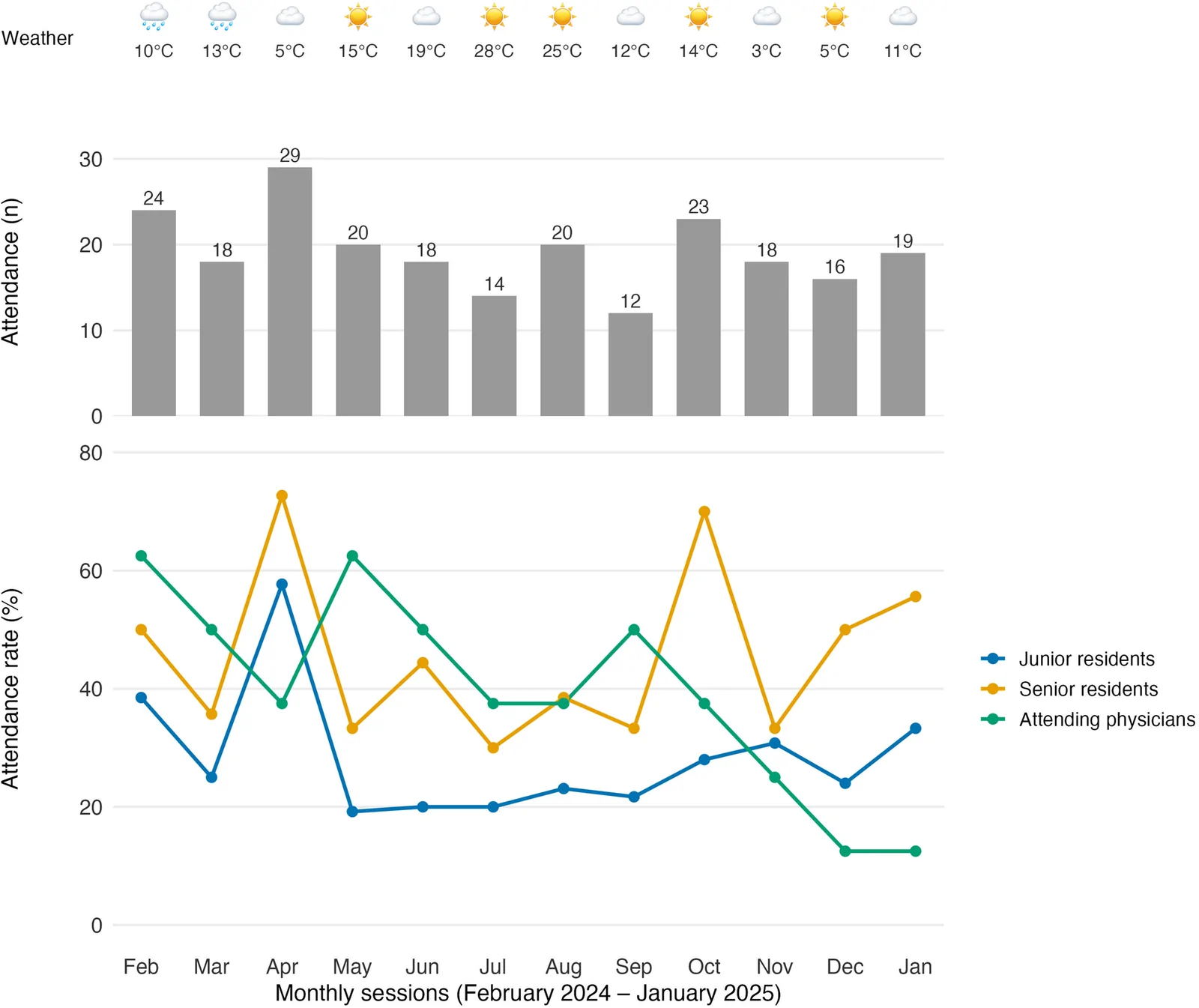
Weather features and attendance rates over the 12 PA sessions. We calculated the attendance rate for each session by dividing the number of attendances at that session by the total number actively scheduled to work in the division on that day.

Supplementary Tables S2, S3 provide detailed data and attendance rates among the target population. Overall, junior residents’ attendance rate was just under 30%, while that of senior residents was just under 50%.

Across all sessions, two thirds of participants learned about the PA program through word-of-mouth from colleagues. About 40%–50% heard about the sessions via oral announcements during other postgraduate trainings, the week-long online announcement on the hospital intranet's Internal Medicine Division page, or through the weekly seminar program sent to residents. Only 20% became aware of the sessions through email reminders sent two days prior to all the residents.

### Acceptability of the PA program

Among participants, the overall mean response rate to the 12 anonymous post-session surveys was 54.1% (Supplementary Table S4). Overall, 97.3% of respondents rated the organization of the activity as very satisfactory, while only 0.5% felt it needed improvement. A total of 95.7% found the activity beneficial, and group-based PA was perceived as highly motivating by 87.7% of respondents. Finally, 84.1% of respondents reported feeling motivated to increase their PA after the session.

### Awareness, participation and impact of the PA program among residents

The anonymous survey conducted after eight months had a response rate of 66.0% (35/53), with 74.3% (26/35) of responses fully completed. As none of the questions were mandatory, denominators differ across items and are reported for each question. There was a high awareness of the PA program among the target population, with 88.6% (31/35) of respondents having heard of it (Figure 3). If all non-respondents were assumed to be unaware, awareness would fall to 58.5% (31/53) of the target population. As in the post-session surveys, word-of-mouth through colleagues was the most common source of awareness (Supplementary Figure S1).

**Figure 3:**
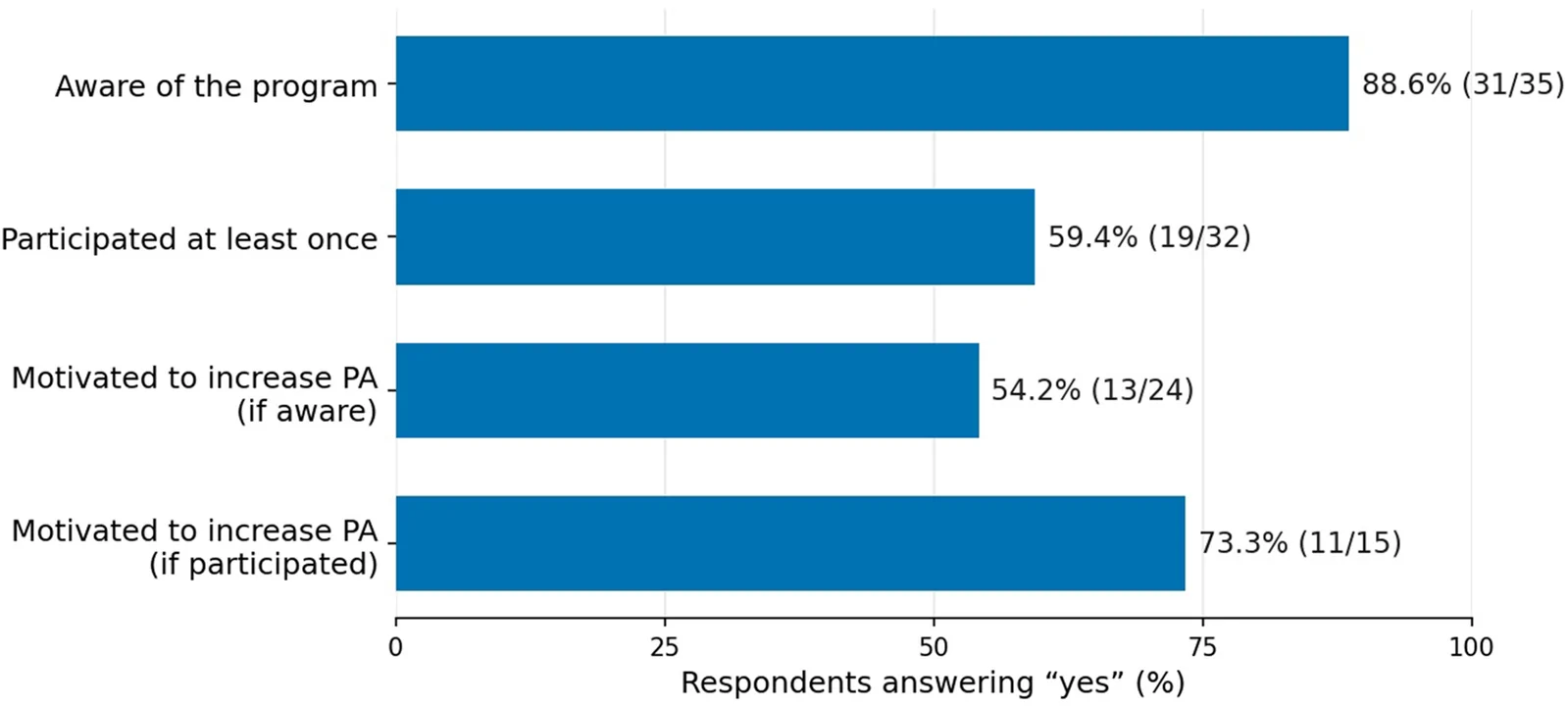
Program awareness, participation (penetration), and impact at 8 months. Awareness was defined as the number of respondents answering “yes” to the question “Have you ever heard of *MIAuSport*?” divided by the total completed surveys for that question (31/35). Participation was defined as the number of respondents reporting at least one participation to the question “How many times have you participated?” divided by the total completed surveys for that question (19/32). Among the 24 respondents who were aware of MIAuSport and answered this question, 13 (54%) answered “yes” to “Have the *MIAuSport* initiatives made you want to increase your daily physical activity?”. Among the 15 respondents who participated at least once and answered this question, 11 (73%) answered “yes” to the same question. None of the questions were mandatory; denominators therefore differ across items.

### Penetration of the PA program

Penetration was moderate, with 59.4% (19/32) of respondents to this question reporting having attended at least one session in the past eight months (Figure 3). Among responders, 75% reported attending one to three sessions during that period (Supplementary Figure S2). Regarding the impact on the residents’ daily PA, 54% (13/24) of responders aware of the PA program reported being motivated to increase their PA, regardless of their participation (Figure 3). This rose to 73% (11/15) among participants. Specifically, non-participants motivated to increase PA reported more frequent participation in activities outside work, while participants showed increased stair use at work.

Among all participants who attended at least one of the 12 sessions, 86% reported engaging in regular PA at least once a week. In the anonymous survey targeting residents, 87% of those who participated in *MIAuSport* reported having a regular PA habit, compared to 82% among those who did not participate.

### Facilitators and barriers for the PA program implementation

Supplementary Tables S5, S6 present participants’ original responses regarding facilitators and barriers.

Suggested improvements to enhance the program included respecting scheduled start and end times, avoiding Friday sessions, and preferably holding sessions mid-week (e.g., Wednesdays between 12:00 and 1:00 or before lunch). Increasing the frequency of sessions to at least twice a month, or even weekly, was proposed to help establish a routine, with some advocating for mandatory participation. Others suggested extending sessions to 60 min and providing designated pager coverage to reduce clinical disruption.

Participants also emphasized the importance of activity variety and proposed several communication improvements: providing clearer explanations at the start of each session, issuing more frequent reminders, increasing publicity, and integrating sessions into electronic calendars to improve visibility. Strengthening group dynamics through greater presence of attendings and setting medium-term goals (such as forming teams to participate in local running events) were also proposed. Finally, favorable weather and a positive atmosphere were recognized as important external facilitators.

Barriers identified by participants included a heavy workload and limited time, especially on Fridays, with some unable to participate due to shift rotations or scheduling conflicts. One participant expressed concern that attending the session would place additional workload on colleagues during that time and prevent leaving work early. Logistical challenges were also mentioned, including the time to shower and change clothes after the sessions. Lack of information and awareness, particularly among new staff or those on rotation further hindered participation. Weather conditions were seen as a potential barrier. Additionally, personal preferences, including dislike of sweating, low motivation for midday exercise, or general disinterest in PA during work hours, further limited engagement for some.

## Discussion

This study evaluated the feasibility and acceptability of *MIAuSport*, a low-cost, workplace-based PA program targeting internal medicine residents in a university hospital setting. Our findings suggest that a structured PA program is both feasible and well-received by residents, even in a demanding clinical environment. This is particularly relevant given the well-documented paradox that, despite high awareness of PA benefits, HCWs continue to exhibit high rates of physical inactivity, often due to organizational and structural barriers (11, 12). Our results reflect these challenges, with some professional groups, such as nurses, unable to attend midday sessions because of shift constraints.

Program awareness at eight months was reported as high among survey respondents, suggesting that the communication strategy was effective. However, this result is likely overestimated by selection bias, as those already aware of the program may have been more inclined to respond. If all non-responders were assumed to be unaware of the program, awareness would drop to less than two-thirds of the target population (58.5%). Despite moderate to high awareness, only 59.4% (19/32) of respondents had attended at least one session, denoting a moderate penetration. This might be explained by the fact that junior residents rotate in different internal medicine services every 3 to 6 months, causing participation discontinuation and underlining the challenge of sustained engagement. Among the three sessions with the highest participation, two coincided with the final month preceding the rotation of junior residents. One possible explanation is that junior residents were more aware of the *MIAuSport* program by the end of their rotations, which may have encouraged their participation. This interpretation, however, requires confirmation over a longer observation period.

The main barriers to participation were organizational, including workload and time constraints, consistent with the literature on workplace PA interventions (28). These results underscore the crucial need for stronger organizational support and integration of PA into the institutional work culture. Stratified attendance rates further highlight the role of senior residents and attendings in motivating their teams, as junior residents and senior resident participation followed similar trends. This suggests that leadership engagement at all levels is critical to promoting such initiatives. The choice between two possible activities also probably enhanced the attendance rate as half of participants chose running and the other half chose walking; further diversification of activities, such as strength training could increase it further. Finally, a change of weekday was considered after the mid-program feedback but could not be implemented during the study period, as the other lunchtime slots remained unavailable.

From a public health perspective, each monthly 40-minute session provided approximately 27% of the 150 weekly minutes of moderate-intensity PA recommended by the WHO for the week in which it took place, corresponding to roughly 6% of the recommended monthly volume (19). While modest, these contributions represent a meaningful step towards increasing weekly PA levels among HCWs.

As previously described in the literature, participants who engaged in the program expressed high satisfaction (29, 30). Most participants reported a perceived benefit from the PA program and three-quarters felt motivated to increase their overall PA, suggesting potential spillover effects beyond the workplace. Such programs, as already shown although not analyzed in this study, could also permit social interaction, increase concentration at work and support broader PA promotion, such as taking the stairs.

This study has several limitations. Participation was voluntary (although formally the postgraduate trainings are mandatory), and subject to self-selection bias, potentially over-representing health-conscious individuals. As a result, satisfaction, perceived benefits, and reported motivation are most plausibly overestimated relative to the full target population. Most data were self-reported, introducing the possibility of recall and social desirability bias. The latter may have been reinforced by the in-person distribution of the post-session survey QR code by program organizers, although responses were anonymous and could be completed at any time. Demographics characteristics were collected at the end of each survey and were not mandatory, resulting in substantial missing values. Because attendance was recorded anonymously without individual identifiers, the number of unique participants and patterns of repeat participation could not be determined; future implementations should consider pseudonymized attendance tracking. Also, due to resident turnover every three months, individual attendance rate over 8 months is difficult to evaluate. The monocentric design limits generalizability, and the absence of objective pre-post fitness or health outcomes precludes conclusions about program effectiveness. Future studies should incorporate objective PA tracking (e.g., accelerometry) to assess baseline activity levels and intervention impact more robustly.

Despite these limitations, this study provides actionable insights for the development of institutional strategies to promote PA among HCWs. Embedding brief, regular PA sessions into working hours appears to be an acceptable and feasible approach to promote PA among HCWs. Improving participation will likely require stronger organizational commitment, visible leadership support, and structural adjustment to enable participation among non-physicians.

In the longer run, such programs may improve not only HCWs’ health but also their credibility and effectiveness in counseling patients about healthy lifestyles. Workplace PA programs have also been shown to prevent work ability deterioration among HCWs in a randomized control trial (18). While our findings are preliminary, they provide a foundation for future trials aimed at measuring health and behavioral benefits of PA among HCWs.

To conclude, *MIAuSport* demonstrates the feasibility and acceptability of implementing a workplace-based PA program in a hospital setting. Although long-term individual engagement could not be assessed and remains a challenge, the program was well received and has potential to be scaled up and strengthened. Future research should focus on objective outcome measures, organizational enablers, and strategies to improve long-term participation among all categories of HCWs.

## Data Availability

The raw data supporting the conclusions of this article can be made available by the authors upon reasonable request.
